# Determinants of routine immunization coverage in Bungudu, Zamfara State, Northern Nigeria, May 2010

**DOI:** 10.11694/pamj.supp.2014.18.1.4149

**Published:** 2014-07-21

**Authors:** Saheed Gidado, Patrick Nguku, Oladayo Biya, Ndadilnasiya Endie Waziri, Abdulaziz Mohammed, Peter Nsubuga, Henry Akpan, Akin Oyemakinde, Abdulsalami Nasidi, Idris Suleman, Emmanuel Abanida, Yusuf Musa, Kabir Sabitu

**Affiliations:** 1 Nigeria Field Epidemiology and Laboratory Training Programme, Abuja, Nigeria; 2 Department of Public Health, Federal Ministry of Health, Abuja, Nigeria; 3 Global Public Health Solutions, Atlanta, GA, USA; 4 Department of Community Medicine, Ahmadu Bello University, Zaria, Nigeria; 5 National Primary Health Care Development Agency, Abuja, Nigeria; 6 Department of Primary Health Care, Ministry of Health, Gusau, Zamfara State, Nigeria

**Keywords:** Immunization, routine immunization, routine immunization coverage, fully immunized child, Nigeria

## Abstract

**Introduction:**

Immunization is a cost-effective public health intervention to reduce morbidity and mortality associated with infectious diseases. The Nigeria Demographic and Health Survey of 2008 indicated that only 5.4% of children aged 12-23 months in Bungudu, Zamfara State were fully immunized. We conducted this study to identify the determinants of routine immunization coverage in this community.

**Methods:**

We conducted a cross-sectional study. We sampled 450 children aged 12-23 months. We interviewed mothers of these children using structured questionnaire to collect data on socio-demographic characteristics, knowledge on immunization, vaccination status of children and reasons for non-vaccination. We defined a fully immunized child as a child who had received one dose of BCG, three doses of oral polio vaccine, three doses of Diptheria-Pertusis-Tetanus vaccine and one dose of measles vaccine by 12 months of age. We performed bivariate analysis and logistic regression using Epi-info software.

**Results:**

The mean age of mothers and children were 27 years (standard error (SE): 0.27 year) and 17 months (SE: 0.8 month) respectively. Seventy nine percent of mothers had no formal education while 84% did not possess satisfactory knowledge on immunization. Only 7.6% of children were fully immunized. Logistic regression showed that possessing satisfactory knowledge (Adjusted OR=18.4, 95% CI=3.6-94.7) and at least secondary education (Adjusted OR=3.6, 95% CI=1.2-10.6) were significantly correlated with full immunization.

**Conclusion:**

The major determinants of immunization coverage were maternal knowledge and educational status. Raising the level of maternal knowledge and increasing maternal literacy level are essential to improve immunization coverage in this community.

## Introduction

Immunization is a proven tool and a cost-effective public health intervention to reduce morbidity and mortality associated with infectious diseases [1]; it is one of the key elements of primary health care [2]. Immunization services are usually delivered via two main strategies namely routine immunization (RI) and supplemental immunization activities (SIAs). RI is the regular provision of immunization services to successive cohorts of infants through the administration of vaccines (antigens) in a scheduled regimen [3]. SIAs are mass campaigns targeting all children in a defined age group with the objective of reaching a high proportion of susceptible individuals [3]. RI services are usually provided at fixed post at the health facility and through outreach to remote and hard-to-reach communities [3, 4].

In 1974, the World Health Organization (WHO) launched the Expanded Programme on Immunization (EPI) [5]. EPI's goals were to ensure that every child received protection against childhood tuberculosis, poliomyelitis, diphtheria, pertusis, tetanus and measles by 1 year of age [4]. In Nigeria, EPI was initiated in 1979 [5]. The country achieved modest progress in immunization coverage during 1980 -1990 [6]. However, the 1990s witnessed a major decline in RI coverage mainly due to the collapse of the Primary Health Care system, poor funding by governments and lack of political commitment and ownership [7]. In 1996, Nigeria's EPI programme was revitalized with renewed government ownership and oversight. Although this led to an increase in immunization coverage, immunization surveys conducted in the country in 2003 and 2006 indicated that the coverage for all the antigens was still below 50% [8, 9].

Of the six geo-political zones in Nigeria, the north-west zone has the worst RI coverage in the country [7–10]. The low RI coverage in this zone has been a major factor for the continuous transmission of wild polio virus and circulating vaccine derived polio-virus (cVDPV) in Nigeria [11]. Among the seven states in north-west Nigeria, Zamfara State has one of the poorest immunization coverage -in 2008, the state recorded a Diptheria-Pertusis-Tetanus (DPT) 3 vaccine coverage of 8.8%, Oral Polio Vaccine (OPV) 3 of 22.8% and measles vaccine coverage of 14.1% [10]. Bungudu represents a typical rural community in Zamfara State and the coverage for RI antigens in this community is below the 80% average required for herd immunity against vaccine preventable diseases (VPDs). The administrative coverage for OPV3 and DPT3 in Bungudu were 22% and 54% respectively in 2006, and 35% and 59% for OPV3 and DPT3 respectively in 2007 [12]. A nation-wide demographic and health survey conducted in 2008 indicated that only 5.4% of children aged 12-23 months in this community were fully immunized [10]. Based on this, we conducted a study to assess the knowledge of mothers (caregivers) on RI, determine the coverage for RI antigens and identify the determinants of full immunization to guide evidenced-based interventions to improve immunization coverage in the community.

## Methods

**Study Setting:** Bungudu is a rural community in Zamfara State, north-west Nigeria. The community has an estimated population of 69, 906 people projected from the 2006 National population census. Majority of the inhabitants are Hausas and are predominantly farmers. RI services in the community are delivered by six primary and one secondary health facilities (all government owned), and through immunization outreach services. The community is divided into three districts and each district has four villages. Altogether, there are 47 settlements in the community; each settlement consists of several households. For the purpose of this study, each settlement constituted a cluster.

**Study Design:** We conducted a community-based cross-sectional study. Our respondents were mothers or caregivers of sampled children. For a child to be eligible for sampling, he or she must have been between 12-23 months old at the time of the study.

**Sample size determination:** We determined the number of children to be sampled using the methods recommended by the World Health Organization (WHO) for Immunization Coverage Cluster Survey [13]. The calculation of the sample size was based on a hypothesized full immunization coverage of 5.4% [9], significance level of 5% -corresponding to a standard normal deviate (z) of 1.96, power of 80%, precision (d) of 3% and design effect (DEFF) of 2. We used the formula: **n = {(z** ^**2**^ **pq/d** ^**2**^ **) x DEFF}** [13] and obtained a minimum sample size of 436 children. However, we sampled equal number of children from each of 30 clusters [13]; thus, 15 children were sampled per cluster giving a total sample size of 450 children.

**Sampling Method:** We employed a two-stage cluster sampling technique to sample eligible children.

*Stage one: Selection of clusters:* At stage one, we selected 30 clusters from the available 47 clusters based on probability proportionate to the size of the population. To select the 30 clusters, the following steps were undertaken: - We randomly listed all the 12 villages in the community, indicating the population size for each village; - We computed the cumulative population by adding the population of the next village on the list to the sum of the population of the previous villages. For example the population of the second village was added to that of the first, the population of the third village was added to the total population sum of the first and second villages and so on; - We then determined the sampling interval by dividing the total population of the community by the number of clusters to be selected: 69,906/30 = 2,330; - We selected a random number using tables of random numbers. The selected random number was 1359; - To determine the village in which cluster one was located, we identified the first village listed in which the cumulative population equals to or exceed the random number (1359). To determine the village where cluster two was located, we added the sampling interval to the random number (2330 + 1359 = 3689) and identified the village whose cumulative population contained this number (3689). To identify the villages where subsequent clusters (clusters three to thirty) were located, we kept on adding the sampling interval to the “preceding sum (running total) of the sampling interval and random number” and locating the village whose cumulative population contained this number (Table 1).

**Table 1:** Selection of study clusters in different villages in Bungudu, Zamfara State, Northern Nigeria, May 2010

Villages	Population	Cumulative Population	Cluster numbers
Sabongari Gidan Dangwari	3500	3500	1
Durumbu	7480	10980	2, 3,4, 5
Sarkin Diya Kasharuwa	7601	18581	6, 7, 8
Sabongari Damba	5800	24381	9,10,11
Sarkin Fada	4132	28513	12,13
Yanruwa	4300	32813	14,15
Nasarawa	2851	35664	16
Dan Galadima	4117	39781	17,18
Tudun Saye	7060	46841	19, 20, 21
Sabongari Yartukunya	4565	51406	22, 23
Tudun Bungudu	6800	58206	24, 25
Galadima	11700	69906	26, 27, 28, 29, 30

*Stage two:* Selection of households: At stage two, we selected 15 households from each of the 30 clusters selected at stage one. The first household in each cluster was selected randomly using table of random numbers. Subsequent households were selected contiguously in the right direction until the number of households for that cluster was completed. From each selected household, one eligible child was selected. If a selected household had more than one eligible child, only one was randomly selected. If a selected household had no eligible child, the next contiguous household was visited and one eligible child selected.

**Data Collection:** Data for the study were collected by female data collectors using structured interviewer-administered questionnaire. We collected data on socio-demographic characteristics of mothers and children, knowledge and attitudes of mothers regarding RI and VPDs, vaccination status of children and reasons for non-vaccination.

**Grading of knowledge of respondents:** To assess the knowledge of mothers, we scored their responses to five questions on various aspects of RI and VPDs. Each correct response was scored one point while each wrong response was scored zero. Mothers who scored 2 points and below were graded as having poor knowledge while those who scored 3 points and above were graded as having satisfactory knowledge[14]. To reduce the possibility of guessing by the mothers, we asked only open-ended questions to assess the level knowledge [15].

**Validity of RI antigens:** For any antigen administered to a child to be considered valid, that antigen must have been administered at the recommended age; and for multiple dose antigens, not less than 4 weeks interval between the doses [13, 14]. We considered a child's BCG vaccine valid, if a scar was present irrespective of whether the vaccination was recorded on the card or obtained by history. BCG vaccination recorded on the card but without a scar was also considered valid [13].

**Vaccination status of children:** Based on the type and doses of valid RI antigens received, we categorized the children as fully immunized, partially immunized, or un-immunized. We defined these categories of vaccination status as follows: - Fully immunized child: a child who had received one dose of BCG, three doses of OPV (excluding OPV given at birth), three doses of DPT vaccine and one dose of measles vaccine by 12 months of age [9, 10]; - Partially immunized child: a child who missed at least any one of the above doses; - Un-immunized child: a child who had not received any vaccine by 12 months of age.

**Data processing and analysis:** We reviewed all completed questionnaires prior to electronic data entry. We performed double data entry to minimize errors. We conducted univariate analysis to obtain frequency and proportions, and bivariate analysis to identify factors that determine full immunization status. We used the chi-square test to determine statistical significance; p-value of less than 0.05 was considered statistically significant. We created a logistic regression model for factors that were significant at bivariate analysis. Data analysis was performed using Epi-info software version 3.5.1.

**Ethical considerations:** We obtained ethical clearance for the study from the ethical committee of Ahmadu Bello University, Zaria, Nigeria. Permission to conduct the study was also granted by the Zamfara State Ministry of Health, Gusau. Informed consent was obtained from each respondent. Confidentiality of the respondents was assured and maintained during and after the study.

## Results

**Socio-demographic characteristics of mothers and children:** The mean age of mothers was 27 years (standard error (SE) = 0.27 year). The age ranged from 15 to 44 years. Thirty one percent of mothers were between 20 to 24 years old. All the mothers (100%) practiced Islam, 79% had no formal education, 81% were traders, and 98% were married. The mean age of the children was 17 months (SE= 0.8 month); the age ranged from 12 to 23 months. Fifty two percent of the children were males (Table 2).

**Table 2:** Socio-demographic characteristics of respondents in a study to identify the determinants of routine immunization coverage in Bungudu, Zamfara State, Northern Nigeria, May 2010

Socio-demographic characteristics (n=450)	Number of respondents	Proportion (%)
**Age distribution (in years)**		
15 – 19	17	3.8
20 – 24	138	30.7
25 – 29	122	27.1
30 – 34	109	24.2
35 – 39	45	10.0
40 – 44	19	4.2
**Religion**		
Islam	450	100.0
**Highest level of formal education**		
None	355	78.9
Primary	50	11.1
Secondary	35	7.8
Post-secondary	10	2.2
**Occupation**		
Trader	363	80.7
Housewife	64	14.2
Tailor	9	2.0
Teacher	8	1.8
Health worker	4	0.9
Civil servant	2	0.4
**Marital status**		
Married	441	98.0
Separated/Divorced	5	1.1
Widowed	4	0.9

**Knowledge and attitudes on RI and VPDs:** Forty four percent of the mothers knew the correct purpose of childhood immunization, 20% knew the timing of first RI visit, 14% knew the timing of the second visit while 16% knew the timing of the last visit; only 12% knew the correct number of visits to health facility to complete RI. Sixty one percent mentioned measles while 50% mentioned poliomyelitis as VPDs. Malaria and diarrhea diseases were also mentioned as VPDs by 12% and 6% of mothers respectively. The commonest symptoms of VPDs recalled by the mothers were fever (57%), followed by cough (48%), skin rash (34%) and paralysis (19%). Seventy nine percent of the mothers believed that immunization is beneficial to children, 81% believed that immunization is safe, while 66% believed that immunization is very effective in preventing VPDs in children. However, 14% of mothers believed that immunization can cause infertility later in the life of children, while 63% believed that immunization prevents all childhood diseases.

**Grading of knowledge:** Forty three percent of mothers had a knowledge score of zero, 28% scored one point while 12% scored 2 points. Five percent of mothers had a score of 3 points; another 5% scored 4 points while 7% scored 5 points. Based on the scores, 84% possessed poor knowledge (score of 0 - 2 points) while 16% possessed satisfactory knowledge (score of 3 - 5 points). High education level was significantly associated with satisfactory knowledge - 46% of mothers whose knowledge were satisfactory possessed high education level (secondary/post-secondary) (p-value: < 0.05).

**Coverage for RI antigens:** The coverage for all RI antigens obtained by both maternal history and immunization card is shown in Table 3.

**Table 3:** Vaccination coverage for routine immunization antigens in Bungudu, Zamfara State, Northern Nigeria, May 2010

RI Antigens	Coverage by maternal history (%); n = 450	Coverage by immunization card (%); n = 450
**Antigens administered at birth**		
BCG	80 (17.8)	40 (8.9)
OPV 0	89 (19.8)	41 (9.1)
HBV 1	84 (18.7)	41 (9.1)
**Antigens administered at 6 weeks**		
OPV 1	73 (16.2)	38 (8.4)
DPT 1	76 (16.9)	41 (9.1)
HBV 2	66 (14.7)	38 (8.4)
**Antigens administered at 10 weeks**		
OPV 2	55 (12.2)	33 (7.3)
DPT 2	60 (13.3)	35 (7.8)
**Antigens administered at 14 weeks**		
OPV 3	41 (9.1)	26 (5.8)
DPT 3	43 (9.6)	27 (6.0)
HBV 3	44 (9.8)	28 (6.2)
**Antigens administered at 9 months**		
Measles	68 (15.1)	24 (5.3)
Yellow fever	35 (7.8)	22 (4.9)

For all antigens, coverage obtained by maternal history was higher than coverage obtained by immunization card. The proportion of children vaccinated with OPV 0 (19.8%), HBV 1 (18.7%) and BCG (17.8%) - all given at birth, were more than the proportion of children vaccinated with antigens given at other times. According to maternal history 7.6% of the children had been fully immunized, 18.9% were partially immunized, while 73.6% were un-immunized. However, according to the immunization cards 4.7% of the children had been fully immunized while 4.9% were partially immunized.

**Figure 1:**
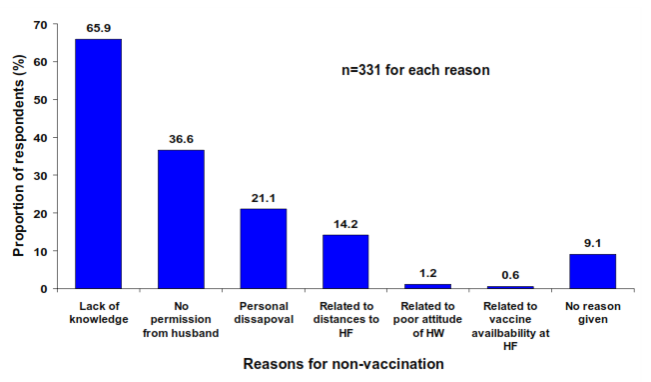
Reasons for non-vaccination of children in Bungudu, Zamfara State, Northern Nigeria, May 2010

**Reasons for non-vaccination:** The reasons given by the mothers for non-vaccination of their children are shown in Figure 1.

Sixty six percent of mothers who had never vaccinated their children gave reasons relating to lack of knowledge on RI for non-vaccination of their children, 37% gave reasons relating to lack of permission from husband. Nine percent gave no reason for non-vaccination of their children.

**Determinants of full immunization:** Five factors were significantly associated with full immunization at bivariate analysis: possessing satisfactory level of knowledge on RI, possessing at least secondary education, receiving ante-natal care, having received information on RI in the 12 months preceding the study, and delivery at health facility (Table 4). Of these factors, possessing satisfactory level of knowledge on RI (p-value: < 0.05) and possessing at least secondary education (p-value: < 0.05) remained as the only independent determinants of full immunization after performing logistic regression (Table 5).

**Table 4:** Bivariate analysis of factors associated with full immunization in Bungudu, Zamfara State, Northern Nigeria, May 2010

Factors	Partially/Un-immunized (%)	Fully immunized (%)	Chi-square (X^2^)
**Highest educational Level**			
None/Primary	392 (94.2)	13 (38.2)	
Secondary/post-secondary	24 (5.8)	21 (61.8)	**103.4**
**Knowledge grade**			
Poor	374 (89.9)	2 (5.9)	
Satisfactory	42 (10.1)	32 (94.1)	**155.4**
**Received ANC**			
No	324 (77.9)	0 (0)	
Yes	92 (22.1)	34 (100.0)	**90.8**
**Received RI information in last 12 months**			
No	111 (26.7)	1 (2.9)	
Yes	305 (73.3)	33 (97.1)	**8.2**
**Place of delivery of index child**			
Home	354 (85.1)	4 (11.8)	
Health facility	62 (14.9)	30 (88.2)	**99.5**

**Table 5:** Logistic regression analysis of factors associated with full immunization in Bungudu, Zamfara State, Northern Nigeria, May 2010

Factors	Odds Ratio	95% C.I.	Coefficient	S.E.	P-value
**Education level**<secondary/ ≥ secondary	**3.63**	**1.24-10.57**	1.29	0.55	**0.0183**
**Knowledge grade** Poor/satisfactory	**18.39**	**3.57-94.70**	2.91	0.84	**0.0005**
**Place of delivery** home/health facility	2.70	0.73-10.00	0.99	0.67	0.1374
**Access to RI information** no/yes	0.51	0.03-8.72	-0.67	1.44	0.6443
**Attendance of ANC** no/yes	1.75	0.00-1.02	12.6324	1.53	0.9038

## Discussion

We found that the majority of mothers in our study in a rural community in Zamfara State possessed poor knowledge on RI and VPDs. We found that high education level was significantly associated with satisfactory knowledge. Although, the attitude of mothers towards immunization was generally positive, some believed that immunization can cause infertility in children. We obtained very low coverage for all RI antigens. Less than 10% of the children were fully immunized. Almost three out of every four mothers had never vaccinated their children. We found that possessing satisfactory knowledge on RI and possessing high education level were the independent determinants of full immunization in this community.

Majority of mothers in our study community possessed poor knowledge on RI, similar to findings obtained in North India [16]. The poor knowledge of mothers in our study may be partly, attributed to the low level of education in this community. We established that mothers with low educational level were less knowledgeable on RI compared to those with high educational level. Education has been described as the root of knowledge. It is expected that mothers with high education level ought to understand scientific information more easily than those with low educational level. This finding is consistent with that obtained in Edo, Southern Nigeria and Istanbul, Turkey [14, 17]. Measles and poliomyelitis were the most common VPDs recalled by mothers in our study, similar to findings obtained in some states in Nigeria [18]. Both diseases are on the center stage of both national and global public health activities, while measles is targeted for elimination in Nigeria, poliomyelitis is targeted for global eradication [19]. Most mothers especially, in northern Nigeria are familiar with the characteristic maculo-papular rash associated with measles infection [20]. In addition, the repeated polio SIAs in Nigeria has popularized poliomyelitis especially in rural communities in northern Nigeria.

Fourteen percent of mothers in our study community believed that immunization can cause infertility in children. This finding readily brings to mind the event that led to the suspension of immunization activities in northern Nigeria between 2003 and 2005. During this period, OPV was erroneously perceived to possess anti-fertility constituents [21, 22]. The widespread mis-conception resulted in poor acceptability and outright suspension of immunization activities in several northern states, perhaps due to the decision of parents to defy purported plots of the western world to reduce the Nigeria's population. This period witnessed a major set-back for immunization activities in Nigeria as both RI and SIAs dipped profoundly. Our study provides rationale to scale up public enlightenment and social mobilization activities and engagement of religious, traditional and political leaders to correct this misconception.

The coverage we obtained for all antigens in our study was lower than that reported by several other researchers [14, 16, 17, 23]. The proportion of children found to be fully immunized in our study was lower than findings in Edo, Southern Nigeria, Brazil and Turkey [14, 17, 23]. However, it is comparable to figure obtained by the NDHS of 2008 [10]. The 9.1% OPV3 coverage we obtained in our study has a great implication for the global polio eradication initiative; this OPV3 coverage is far below the 80% recommended by the WHO for polio eradication [19], and creates a substantial population gap - a key risk factor for the emergence and circulation of cVDPV [24, 25]. Similarly, the 15.1% coverage we obtained for measles vaccine is lower than the 90% recommended by the WHO/ UNICEF strategic plan for measles morbidity and mortality reduction [3].

We found that satisfactory maternal knowledge on RI is an independent determinant of full immunization in this community. This finding is consistent with those of other researchers [14, 26]. As expected, knowledge regarding the benefit and schedule of RI is a powerful tool that positively influences a mother's decision to fully immunize her child. However, educational level, which we found to be significantly associated with knowledge, is very low in this community. This correlates with our finding that only 7.6% of mothers had fully immunized their children. Furthermore, findings from our study indicated that high education level was an independent determinant of full immunization similar to findings obtained in Edo State, Southern Nigeria and Turkey [14, 17]. However, a study conducted in an urban area of Brazil demonstrated that maternal literacy was not associated with full immunization [23]. This contrasting finding in Brazil may possibly, be due to the urban setting of the study and the recruitment of study participants from the health facilities rather than the community. The independent effect of high education level on full immunization demonstrated by our study highlights the need for inter-sectoral collaboration between the health and education sectors to improve immunization coverage in this community.

The interpretation and generalization of the findings from our study is subject to three limitations. Firstly, we did not explore factors related to immunization service delivery including vaccine availability, health care personnel and logistics. Secondly, we could not verify the information provided by the respondents regarding the antigens received by their children. We tried our best to describe the site, dose and timing of the antigens to obtain accurate information. Finally, our study was limited in geographical scope. Although, our study community is a good prototype of rural communities in northern Nigeria, we acknowledged that conducting this study in the entire Zamfara State or northern Nigeria could have produced different results. However, we used an absolute precision of 3% instead of 5%-10% to achieve a sufficiently large sample size to increase the precision and allows for generalization of our findings, at least in Zamfara State.

## Conclusion

In conclusion, the maternal knowledge and literacy level in this community is very low. Uptake of RI antigens was also, generally very low. Poor maternal knowledge and low level of education independently, determine full childhood immunization in the community. The community, supported by the State Ministry of Health and the State Ministry of Information should embark on focused public enlightenment and health education activities on the benefits, schedule and doses of RI, targeting both mothers and fathers to improve the level of knowledge and correct misconceptions regarding some aspects of RI. Inter-sectoral collaborations between the health and education sector should be strengthened. In this light, the State Ministry of Education and other relevant partner agencies should support the organization of flexible adult educational classes as well as the enrolment of the girl child into primary and secondary school.
